# Co-Creating Safe Suicide Reporting Guidelines with Journalists

**DOI:** 10.1192/j.eurpsy.2026.12084

**Published:** 2026-07-31

**Authors:** S. A. Geegan, C. Rhein

**Affiliations:** Integrated Strategic Communication, University of Kentucky, Lexington, United States

## Abstract

**Introduction:**

How media report on suicide can influence population-level risk, help‐seeking, and stigma. Evidence‐based safe messaging guidelines aim to reduce contagion effects and promote responsible coverage. However, compliance with these guidelines has been uneven. Understanding how journalists interpret guideline recommendations—or why they deviate—is critical to bridging the gap between guideline availability and real-world practice.

**Objectives:**

This study examined how journalists interpret and apply suicide media guidelines, with a focus on barriers and facilitators to their use in the newsroom. Using Kentucky, USA as a case context, the investigation explored organizational and editorial factors shaping suicide coverage. Importantly, the research team engaged journalists in a co-creation process. By centering journalists’ perspectives, the study generated insights to inform guideline development and promotion.

**Methods:**

Semi-structured interviews were conducted with journalists (print, online, and broadcast; N=12). Purposive sampling was used to capture variation in newsroom size, audience reach, and editorial structure. Beyond exploring knowledge, attitudes, and newsroom processes related to suicide coverage, the study incorporated a co-creation component. Journalists were presented with an initial draft of reporting guidelines (developed in collaboration with suicide experts) and invited to react, critique, and propose edits. Feedback from journalists was then systematically integrated into subsequent revisions, with changes focused on word choice and tone. This process resulted in a final, co-created document. Interviews were audio-recorded, transcribed verbatim, and analyzed thematically to identify patterns and contextual influences.

**Results:**

Journalists frequently noted that they had received little to no formal training on how to report on suicide. Most participants were unfamiliar with organizations such as NAMI that provide safe messaging guidelines but emphasized the value of such a resource, particularly for its specificity in what to avoid. Participants underscored the importance of framing, favoring guidelines positioned as evidence-based recommendations rather than rigid directives. For example, they were attentive to wording that avoided “do this/don’t do that” rules, which they viewed as undermining their professional autonomy. Finally, media guidelines were perceived as most useful in digital form, to be circulated electronically by editors and available on demand.

**Conclusions:**

Through collaboration with journalists, the study produced a revised guidelines document infused with feedback while maintaining fidelity to evidence-based recommendations. The resource is now positioned for publication with endorsement from the Kentucky Press Association and the state Department for Behavioral Health, reflecting a model of co-created guidance that respects journalistic independence while advancing public health goals.

**Disclosure of Interest:**

None Declared

